# The promising role of miRNAs in radioresistance and chemoresistance of nasopharyngeal carcinoma

**DOI:** 10.3389/fonc.2024.1299249

**Published:** 2024-02-28

**Authors:** Haoyuan Xu, Wanpeng Li, Dehui Wang

**Affiliations:** Department of Otolaryngology - Head and Neck Surgery, Affiliated Eye, Ear, Nose, and Throat Hospital, Fudan University, Shanghai, China

**Keywords:** nasopharyngeal carcinoma, radio resistance, chemoresistance, microRNA, role

## Abstract

Nasopharyngeal carcinoma (NPC) is a malignant epithelial tumor that develops in the nasopharynx. It has a distinct ethnic and geographical distribution, and emerging evidence suggests that it is an ecological disease. Most patients respond well to radiation combined with chemotherapy as the primary treatment for NPC. However, some patients will eventually develop radio resistance and chemoresistance, resulting in recurrence and metastasis, which is a primary cause of poor prognosis. The processes underlying radio resistance and chemoresistance in NPC are complex and unknown. MicroRNAs (miRNAs) are endogenic non-coding RNA molecules. They play a role in a variety of cell functions as well as development of disease such as cancer. There has been considerable data demonstrating the existence of numerous aberrant miRNAs in cancer tissues, cells, and biofluids, which indicates the importance of studying the influence of miRNAs on NPC. Therefore, this review comprehensively analyzes the elaborate mechanisms of miRNAs affecting the radio resistance and chemoresistance of NPC. Multiple tumor-specific miRNAs can be employed as therapeutic and prognostic biological indicators.

## 1 Introduction

Nasopharyngeal carcinoma (NPC) is a type of epithelial cancer that originates from the mucous lining of the nasopharynx. Featuring prominent differences in regional distribution, NPC is most commonly observed in East and South Asia, North America, and Northern Europe (1–3). According to the different pathological features, NPC can be categorized into three types:. nonkeratinizing and keratinizing squamous cell carcinoma, and basaloid squamous cell carcinoma. The nonkeratinizing type of NPC can be further subdivided into differentiated carcinoma and undifferentiated carcinoma. It has been reported that 95% of NPC cases occur in endemic regions and are strongly associated with Epstein-Barr virus (EBV) infection (4–6). In the past, NPC is defined as an inherited disease with varying degrees of intertumor and intratumor heterogeneity (7). Recently, some academics are arguing that the nature of NPC is an ecological disease: a multidimensional spatiotemporal “unity of ecology and evolution” pathological ecosystem, which provides a novel theoretical framework and paradigm for understanding complex tumor causal processes, as well as probable preventive and therapeutic regimens for patients (8). Currently, tremendous progress has been made in the development of NPC therapies. As intensity-modulated radiotherapy (IMRT) is being utilized more extensively and chemotherapy regimens such as concurrent therapy, induction therapy, and adjuvant therapy are being continuously optimized, the survival rate of patients with NPC has increased, and drug toxicity has decreased. Although treatment protocols have improved, do novo metastases occur in approximately 5-11% of NPC patients. Studies have reported that 15-30% of NPC patients with locally advanced disease experience dissemination or local relapse after receiving local treatment (9, 10). The resistance of cancer cells to chemotherapy or radiotherapy, either as an inherent or developed feature, greatly contributes to the metastatic lesion and recurrence of NPC.

MicroRNAs (miRNAs) are a type of endogenous noncoding RNA that generally contain 22–25 nucleotides (11, 12). miRNAs most commonly exert their function by serving as posttranscriptional repressors. When miRNAs bind to Argonaute (AGO) proteins, an RNA-induced silencing complex (RISC or miRISC) targets particular mRNA transcripts. To date, convincing data have shown that each of the recognized hallmarks of cancer, including NPC, is subject to miRNA-mediated regulation (13–15). Ionizing radiation and chemotherapy can modulate sensitivity or resistance by inhibiting or inducing the expression of miRNAs.

Several scholars have identified and demonstrated the impact of miRNAs in adjusting the differential expression of functional genes on cell proliferation, invasion, apoptosis, migration, and even novel phenotypes in NPC against the background of radiation resistance and chemotherapy resistance (16–19). Therefore, further exploration and validation of new miRNAs may lead to the identification of vital biomarkers or predictors for identifying radioresistant or chemo resistant patients in the clinic. In this study, we updated and classified abundant miRNAs associated with radiotherapy or chemotherapy response in NPC via a systematic review. Additionally, we investigated detailed mechanisms of miRNAs affecting the radio resistance and chemoresistance of NPC.

## 2 Methods

**Figure 1:**
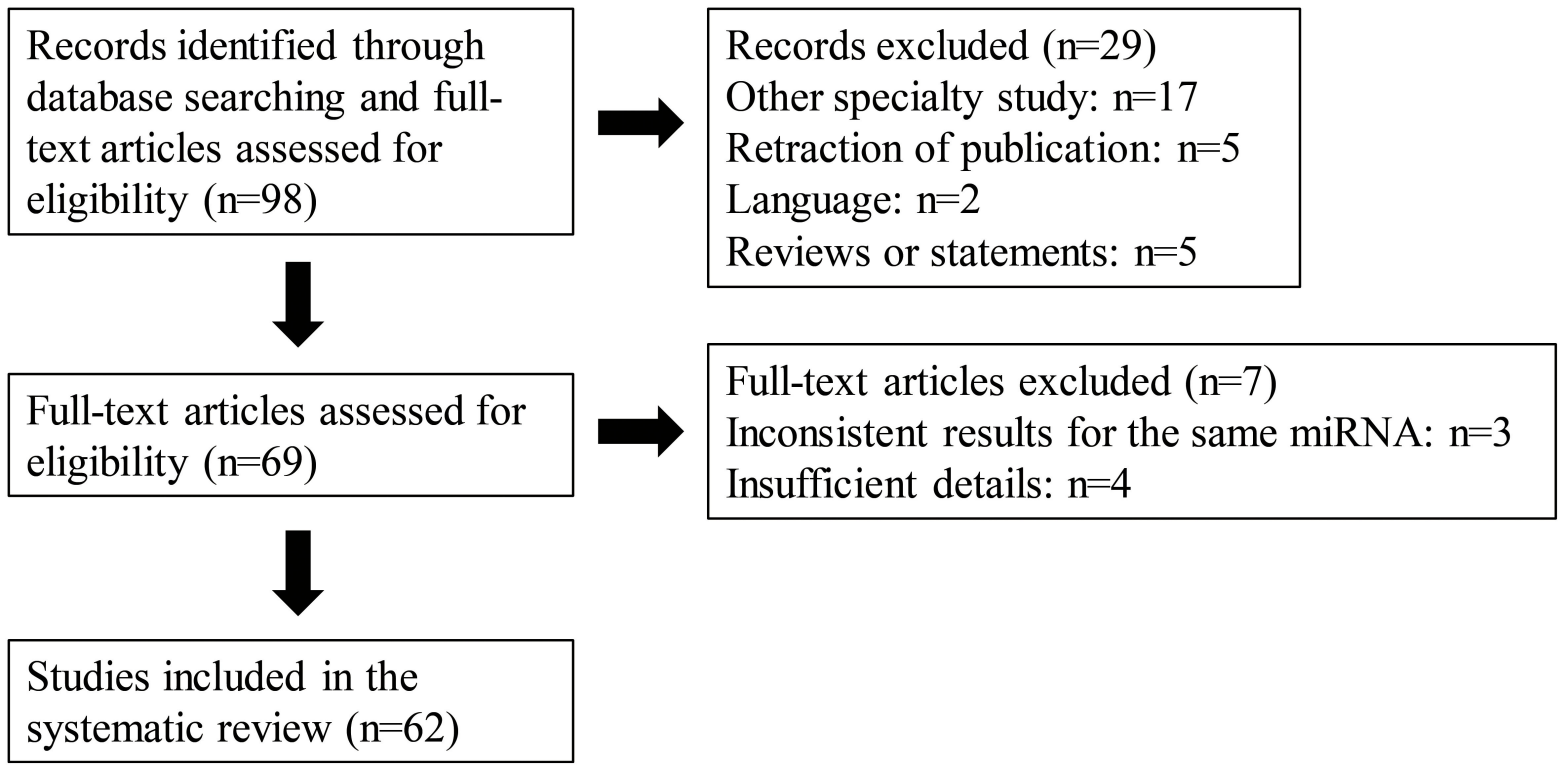
A PRISMA flow diagram presenting the screening process, and the number of included studies with reasons of exclusion.

In this study, we searched for all related English language articles in the NPC field from the PubMed database published between 2017 and 2023. The key words utilized for the research included “nasopharyngeal carcinoma,” “miRNA,” “radiation”, “radio resistance”, “chemotherapy” and “chemoresistance”. Our study comprehensively analyzed the findings of identified publications. Studies of miRNAs from different sources, namely cells, tissues, serum, and exosomes, were all included. The details of the screening process and the number of included studies with reasons of exclusion are shown in the flow chart (**Figure 1**). As illustrated in the flow chart, 62 studies met the inclusion criteria for the final systematic review.

## 3 Results

### 3.1 Overview of miRNA biogenesis and functions

**Figure 2:**
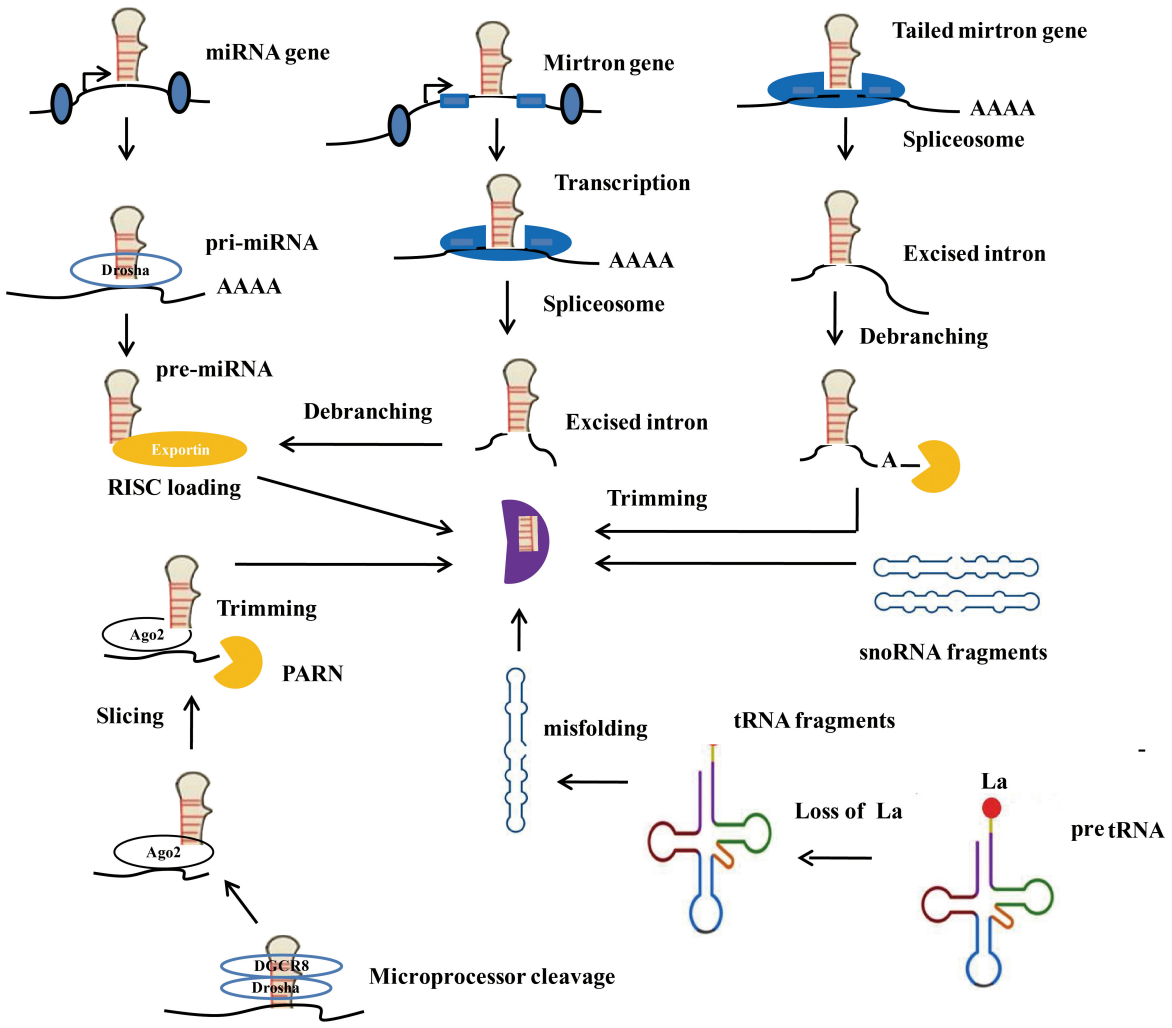
Canonical and Non-canonical microRNA biogenesis. Ago2, Argonaute 2; RISC, RNA-induced silencing complex; PARN, Poly (A) - specific ribonuclease.

Although the specific mechanism of miRNA biogenesis is still unclear, two widely accepted approaches to miRNA biogenesis are known as the classical pathway and the nonclassical pathway, as shown in **Figure 2**. In the classical pathway, biogenesis originates from the nucleus, which synthesizes pri-miRNAs containing one or more hairpins. The nuclear microprocessor complex subsequently processes pri-miRNAs to form pre-miRNAs. After pre-miRNA is conveyed to the cytoplasm and processed by Dicer, mature miRNAs are generated (20). Next, the mature miRNAs bind to the AGO protein to produce the RISC. This process encompasses several critical steps, such as the transcription of miRNAs, the regulation of miRNAs through Dicer and Drosha, and the loading of RISC. In the case of the nonclassical pathway, miRNAs do not need to be processed through Dicer or Drosha. miRNAs, independent of the microprocessor, contain mirtrons and tailed mirtrons. All the mirtrons are produced through splicing and subsequent lariat debranching. As representative microprocessor-independent miRNAs, mirtrons are processed by the nuclear spliceosome first, folded to form short hairpins, and finally access the miRNA pathway to the pre-miRNA phase. The above process explains why the mirtrons escape from cleavage by the microprocessor (21). In addition, other types of endogenous noncoding RNAs, including tRNAs (22), small nucleolar RNAs (23), and small hairpin RNAs (24), can also serve as substrates for nonclassical miRNA biogenesis. Furthermore, research has shown complex factors such as RNA-binding proteins (RBPs), enzymes, and hypoxia can adjust the process of miRNA biogenesis. Overall, the biogenesis of miRNAs and the underlying regulatory mechanisms have not been fully elucidated. More research contributes to understanding the biogenesis of miRNAs in depth.

The most basic function of miRNAs is to serve as the posttranscriptional repressors of gene expression. The combination of miRNAs and AGO proteins can generate a RISC or miRISC, which can target particular mRNA transcripts. There has been additional evidence proving the occurrence of diverse new mechanisms involving miRNAs. miRNAs can influence gene expression by regulating the transcription and epigenetic state of gene enhancers and promoters located within the nucleus (25) and by targeting transcripts encoded by mitochondria (26, 27). For instance, after nuclear miR-181c is transferred to cardiomyocytes mitochondria, it can contribute to miRNA-dependent silencing of mt-Co1 (27, 28) Several miRNAs have also been shown directly impact protein function. This effect is achieved through direct biophysical activities and eventually regulates important functions of cardiovascular biology such as the integrity of endothelial cells (miR-126-5p by suppressing caspase 3) (29) or the potential for cardiac action (miR-1-3p by binding to Kir2.1) (30). *In vitro*, miRNAs can also repress targets or attach to cell receptors (such as Toll-like receptors) on receptor cells to promote communication between cells (31).

### 3.2 MiRNA associated with radio resistance

Numerous differentially expressed miRNAs, including EBV-encoded miRNAs, in the serum, exosomes, and tissues of radioresistant NPC patients, and in NPC-resistant cell lines have been identified by high-throughput sequencing, microarray analysis, and bioinformatics. Research has shown a trend toward the expression of approximately fifty new miRNAs, such as miR-150, miR-483-5p and miR-454-3p, in radiation-resistant NPC (19, 32, 33). Furthermore, more than thirty miRNAs, including miR-31-5p, miR-613 and miR-340-3p, have been shown to modulate the radiosensitivity of NPC (34–36).

After radiation treatment, tumor cells undergo DNA strand breaks, leading to cell apoptosis and cell cycle arrest. Increased tumor volume, reduced oxygen availability, and dysregulated genes can result in radiation tolerance in tumor cells. Existing studies have generally focused on the conventional biological function of miRNAs, that is, the function of targeting the inhibition of posttranscriptional oncogenes and tumor suppressors induced by radiotherapy. The radio resistance of NPC is mediated by decreased expression of apoptotic genes, excessive expression of proliferative and antiapoptotic genes, abnormal expression of cell cycle regulatory genes and cell metabolism associated genes, or enhanced expression of genes used to mediate DNA injury and repair (37–39). Taken together, these findings suggest that miRNAs can influence almost all targeted pathways or genes involved in the radio resistance of NPC.

#### 3.2.1 Affecting proliferation and apoptosis

When proliferative genes are activated and expressed excessively, radio resistance occurs. In contrast to these associated with proliferation, if genes related to apoptosis are abnormally expressed, fewer tumor cells are killed under ionizing radiation. Proliferation and apoptosis phenotypes are commonly reported in current studies on NPC radio resistance. The frequently used research methods include the cell counting kit-8 (CCK-8) assay, flow cytometry apoptosis assay, colony formation assay, detection of proliferation- and apoptosis-related markers, and measurement of tumor size and weight *in vivo*.

As reported in previous publications, miR-1253 knockdown can suppress NPC cell viability, accelerate NPC cell apoptosis and enhance the radiosensitivity of NPC. Mechanistically, upstream circFIP1L1 suppressed the inhibitory effect of miR-1253 on EIF4A3 (16). Moreover, miR-7-5p can reduce the radiosensitivity of NPC cells by promoting cell proliferation and accelerating cell death through the upregulation of ENO2 (18). According to clinical research, there is a positive association between the miR-181a level and lymphatic metastasis and late TNM stage. When miR-181a is expressed ectopically, the growth of radioresistant NPC cells is promoted, which has been verified by multiple assays (40). Moreover, the overexpression of miR-222 and miR-210 reduces the cell apoptosis rate by promoting cell viability, colony formation and tumor growth (41, 42). Conversely, miR-31-5p mimics were found to remarkably slow cell proliferation and attenuate radiation resistance by binding to SFN (35). Cytoplasmic miR-452-5p competitively binds to ZNF621 via LINC01140 to increase cell proliferation and reduce apoptosis (43). Peng et al. demonstrated that X-irradiation (IR) can suppress miR-3942-3p expression. When miR-3942-3p inhibitors were used to downregulate miR-3942-3p expression, the activity of NPC cells improved, while apoptosis decreased (44). A previous study showed that miR-124-3p knockdown could facilitate NPC cell growth and diminish apoptosis caused by irradiation. Additionally, miR-124-3p targets LHX2, which activities the Notch pathway to reduce the radiation sensitivity of NPC cells (45). Cellular experiments by Guo et al. revealed that overexpressed miR-29a promoted CNE-2R cells sensitivity to radiation, which was achieved by inhibiting cell activity and accelerating cell death after irradiation. They also found that miR-29a directly targets COL1A1 to improve NPC cells radio resistance (46). Additionally, miR-125a, miR-138-5p and miR-519d are reportedly positively correlated with radiosensitivity (47–49).

#### 3.2.2 Participation in migration, invasion and metastasis

Research has confirmed other functions of miRNAs in regulating radio resistance due to their ability to influence migration, invasion, and metastasis via transwell assays, Boyden chamber invasion assays, wound scratch assays, and the detection of epithelial-mesenchymal transition (EMT) markers, etc.

Zhou et al. demonstrated that miR-BART8-3p can target and repress their PAG1 host genes and consequently facilitate EMT, invasion, and radio resistance-associated metastases in NPC cells (50). Similarly, Yi et al. reported that when miR-194-3p was inhibited, the invasion and migration of NPC cells were repressed. NPC cells radiosensitivity was enhanced, and cell killing was accelerated (51). The overexpression of miR-BART6-5p in patients with NPC has been demonstrated to be significantly correlated with clinical stage, T stage and pre-DNA. Tang et al. demonstrated that Dicer1 expression is increased and invasion of NPC cells is decreased when miR-BART6-5p is downregulated (52). Luciferase activity assays and bioinformatic software have verified the ability of miR-BART4 to suppress PTEN expression and promote aggressiveness while attenuating the radiosensitivity of NPC (53). However, Wang et al. reported that miR-143-5p can modulate HOXA6 to inhibit the invasion or migration of radiation-resistant NPC cells (17). Wei et al. demonstrated that miR-335-5p can modulate the mTOR and p21 signaling pathways, thereby negatively regulating PADI4 and inhibiting the invasion, movement and radiation tolerance of NPC cells (54). The experiments conducted by He et al. illustrated that when miR-182-5p was upregulated, the suppressive effects of BNIP3 on the migration and invasion of 5-8F-resistant cells decreased (55). In addition, Han et al. reported that when miR-203 was upregulated, the migration and proliferation of nasopharyngeal cancer cells were repressed, and tumor growth was also be suppressed by modulating the ERK/JNK signaling pathway (56). Interestingly, the overexpression of exosome-derived miR-34c also reduces NPC cell resistance to radiation, as it targets β-catenin and represses the EMT in NPC (57). Other data also confirmed that miR-9 (58), miR-372 (59), miR-495 (60), miR-206 (61), miR-186 (62), and miR-138-1-3p (63) knockdown significantly induced cell invasion, metastasis and EMT.

#### 3.2.3 DNA repair and cell cycle regulation involvement

Radiation can cleave DNA by directly forming free radicals and water in ionized cells during the radiation process. However, DNA damage after radiation can be repaired through the DNA damage response (DDR) (64), which is a crucial factor in radiation resistance. When histone H2AX is phosphorylated within chromatin to produce γ-H2AX, DNA double-strand breaks (DSBs) are formed (65). Furthermore, research has revealed a close correlation between the cell cycle distribution of tumor cells and radiosensitivity. Cells exhibit different perceptions of radiation damage in each phase of the cell cycle. Cells at the M stage and the G2 stage had the highest radiosensitivity, while cells at the G1 stage and the S stage had weaker radiation sensitivity. As cells are divided and cell DNA separates actively at the G2 and M stages, radiation is prone to causing DNA damage and cell death.

Xie et al. showed that miR-195-3p overexpression impeded the progression of the cell cycle. CDK1 is a target gene of miR-195-3p, and its overexpression can offset the blockage of the cell cycle and increase in radiation sensitivity caused by miR-195-3p overexpression (66). According to another study, miR-17-5p can accelerate cell division and cell cycle damage induced by different doses of radiotherapy via the PTEN/AKT signaling pathway (67). Zhou et al. reported that EBV-miR-BART8-3p can improve the radiation tolerance of NPC cells and inhibit the progression of the cell cycle at the M or G2 stage, revealing its contribution to postradiotherapy DNA repair. Mechanistically, for the first time, this study proposed the critical effect of EBV-miR-BART8-3p on improving NPC cell radiation tolerance by regulating ATR or ATM expression to prevent DSBs (68). In contrast, the authors revealed that miR-29c and miR-124 knockdown can reduce cell radiosensitivity to irradiation and increase the expression of γ-H2AX (69, 70).

#### 3.2.4 Other novel phenotypes

With the deepening understanding of the mechanism of radio resistance, novel phenotypes closely related to radio resistance, including those related to autophagy pathways, metabolism-related targets, methylations, and cancer stemness, have been explored and validated,.

Autophagy is often the main feedback mechanism of cancer cells to radiation and has been extensively studied in preclinical settings. A mechanistic study revealed that miR-340-3p can suppress FKBP5 expression and alleviate cytophagy, thereby improving the radiation sensitivity of NPC cells. This might lead to the identification of a new target for optimizing the effectiveness of radiotherapy in treating NPC (34). Interestingly, exosomal miR-197-3p can suppress AKT/mTOR signaling by activating phosphorylation and blocking autophagy mediated by heat shock 70-kDa protein 5 (HSPA5) (71). However, another study suggested that miR-454-3p can directly target PTPRD, and that STAT3 is directly dephosphorylated, which promotes ATG5 transcription and stimulates autophagy affected by radiotherapy (19).

Mitochondrial dysfunction, increased glycolysis and other abnormal metabolic activities can lead to radio resistance (72, 73). There is a strong correlation between the development of NPC and the expression of hexokinase 2 (HK2), a subclass of kinases. NPC cells acquire energy mainly through glycolysis, not through oxidative phosphorylation of mitochondria. The glycolytic capacity can be enhanced when HK2 is highly expressed. Zhan et al. revealed that the miR-9-5p can effectively suppress tumor cell growth by targeting HK2 through Kyoto Encyclopedia of Genes and Genomes (KEGG) enrichment, Gene Ontology (GO) and protein-protein interaction network (PPI) analyses of differentially expressed genes (DEGs) (74). The authors found that miR-214 could directly target lactotransferrin (LTF) and enhance the radiosensitivity of NPC cells (75). However, miR-150 targets glycogen synthase kinase-3β (GSK3β) to improve NPC cell resistance to radiation. Western blot assays showed that the expression of GSK3β proteins in CNE-2 resistant cells was repressed, and after restoration, the sensitivity of CNE-2-resistant cells to radiotherapy was improved (33).

Other phenotypes also shed light on the selection of treatments and prognostic targets for radiotherapy resistance. Deng et al. demonstrated that miR-613 can decrease TIMP3 methylation and improve the expression of TIMP3 proteins by suppressing DNMT3B. As a result, the STAT1/FOXO1 signaling pathway was suppressed, and NPC cell sensitivity to radiation was enhanced (36). Notably, miR-124, which targets JAMA, can suppress stemness and increase NPC cells sensitivity to radiation both *in vivo* and *in vitro* (76).

### 3.3 MiRNAs associated with chemoresistance

Chemotherapy is considered an adjunctive therapy for the treatment of NPC. For NPC at the middle and late stages, the common practice is to combine radiotherapy and chemotherapy for improved treatment efficacy (77). Although most patients who receive platinum-based chemotherapy have a positive treatment effect, recurrence is always induced as resistance to chemotherapeutic drugs has increased (78). These findings necessitate further exploration of how multidrug resistance occurs in NPC. Recent studies have shown that miRNAs can mediate the growth, migration, invasion, apoptosis, tumor stemness, exosome formation, DNA damage repair, and autophagy of tumor cells, thereby regulating the emergence and development of chemotherapy resistance in patients with NPC. The regulatory changes in chemotherapy sensitivity may be related to miRNAs inhibiting the expression of oncogenes to promote the expression of tumor suppressor genes or suppressing the expression of tumor suppressor genes to accelerate the expression of oncogenes. Therefore, miRNAs with special functions can be utilized as target agents to improve patient prognosis and diminish drug tolerance.

#### 3.3.1 Cisplatin

Prospective, randomized, controlled clinical trials of platinum-based chemotherapy regimens combined with immune or targeted therapy are ongoing. The combined use of cisplatin and gemcitabine induction chemotherapy can prevent micro metastases and prolong the survival of patients with late-stage local NPC (1, 79). However, the underlying mechanisms of recurrence and metastasis after chemotherapy resistance are unclear. Several miRNAs have been found and validated to modulate cisplatin-based chemotherapy sensitivity through classical or nonclassical phenotypes. Proliferation, apoptosis, migration, invasion, and EMT are still commonly studied phenotypes (**Table 1**).

**Table 1:** miRNAs and cisplatin resistance in NPC.

miRNA	Expression	Source	Targets or pathway
miR-454-3p	Down	Cell,Tissue	HOXA11,AKT/mTOR
miR-34c	Down	Cell,Tissue	SOX4
miR-BART7-3p	Up	Cell,Tissue	SMAD7
miR-449b	Up	Cell	TGFβ1
miR-125b	Down	Cell	Bcl-2
miR-26b	Down	Cell	JAG1
miR-302c-5p	Down	Cell	HSP90AA1
miR-106a-5p	Up	Exosome	ARNT2
miR-515-5p	Down	Cerum	IL-25
miR-BART17-5p,miR-BART19-3p	Down	Cell	BRCA1
miR-218-5p	Down	Cell,Tissue	GDPD5
miR-1278	Down	Cell	ATG2B
miR-342-5p	Down	Cell	–
miR-205	Down	Cell	HER3
miR-205-5p	Up	Cell	PTEN,PI3K/AKT
miR-19b-5p	Down	Cell,Tissue	KRAS
miR-296-3p	Down	Cell,Tissue	MK2,Ras/Braf
miR-139-5p	Down	Cell	–
miR-let-7a	Down	Cell,Tissue	–
miR-519d	Down	Cell,Tissue	PDRG1
miR-106a-5p	Down	Cell	SOX4
miR-338-3p	Down	Cell,Tissue	SMAD5,PI3K/ATK
miR-137	Down	Cell	ERRα
miR-BART22	Up	Cell,Tissue	MAP2K4,PI3K/AKT

Yuan et al. revealed that when a miR-125b inhibitor was transfected, cell death and cytotoxicity caused by DDP were reduced, and cisplatin resistance was enhanced (80). Similarly, Shi et al. reported that ectopic overexpression of miR-26b can suppress cell activity and lead to cell death (81). When evaluating the IC_50_ value, Lin et al. determined that miR-515-5p can be sequestered by circNRIP1 to reduce the IC_50_ value of cisplatin by inhibiting posttranscription IL-25 expression (82). At the functional level, miR-218-5p can inhibit NPC cell proliferation, migration and EMT via modulation of the GDPD5/SEC61A1 axis. This process also enhanced the chemosensitivity of NPC cells (83). In addition, miR-34c, which can directly target SOX4, a major regulator of EMT, was downregulated in NPC patients, inducing the upregulation of Snail, ZEB1, CDH2 and SOX2 and the downregulation of CDH1 and claudin-1 *in vitro*. From the perspective of phenotype, suppression of miR-34c can lead to drug resistance to cisplatin, while overexpression of miR-34c can improve NPC cell sensitivity to cisplatin (84). Additionally, miR-139-5p (85), miR-205 (86), miR-296-3p (87), miR-454-3p (88), miR-19b-5p (89), and miR-106a-5p (90) were also studied and found to improve chemotherapy sensitivity or attenuate chemoresistance *in vivo* and *in vitro* via analogous phenotypes.

Recently, tumor stemness, exosomes, and autophagy have been reported to regulate chemotherapeutic sensitivity to cisplatin. Liu et al. reported that EBV-miR-BART22 could facilitate tumor metastasis and stemness while promoting cisplatin tolerance. Cinobufotalin can powerfully restore cisplatin tolerance induced by EBV-miR-BART22 by activating MAP2K4 to fight against the nonmuscle myosin heavy chain IIA/glycogen synthase 3β/β-catenin pathway (91). Cai et al. reported for the first time the effect of EBV-miR-BART7-3p on promoting tumor sphere growth and increasing the expression of stemness markers at different research levels (92). However, miR-302c-5p can suppress drug resistance to cisplatin and inhibit stem cell properties, as determined by means of a sphere formation assay and detection of the expression of stemness markers (93). Initially, Li et al. compared the exosomal miR-106a-5p level in the serum of patients, and reported that last-cycle patients with chemotherapy resistance had higher exosomal miR-106a-5p levels than first-cycle patients without chemotherapy resistance. In addition, exosomal miR-106a-5p promoted NPC cell proliferation. The main reason is that exosomal miR-106a-5p targets ARNT2 and consequently induces AKT phosphorylation. In addition to enhancing cell proliferation, exosomal miR-106a-5p can also reduce cell death and control the generation of tumors (94). Zhao et al. suggested that a decreased survival rate and a nonideal response to chemotherapy are associated with a reduction in miR-1278. According to the results of the CCK-8 assay, which included a negative control group, excessive miR-1278 expression in NPC cells led to a reduction in growth and a decrease in the IC_50_ of DDP. Additionally, DDP resistance and autophagy inhibition related to miR-1278 can be reversed when ATG2B is highly expressed (95). Interestingly, a novel mechanistic study demonstrated that when TGFβ1 was reduced, additional pro-TGFβ1 was activated and cisplatin tolerance caused by TGFβ1 was enhanced. Therefore, excessive miR-449b overexpression leads to a reduction in TGFβI, which further disrupts the balance between pro-TGFβ1 and TGFβ1. This findings highlights a new mechanism of resistance to chemotherapy in NPC patients (96).

#### 3.3.2 Paclitaxel

Paclitaxel is a common combination drug for NPC chemotherapy. In previous studies, the combination of paclitaxel and carboplatin was shown to have good toxicity and potential for large-scale application in clinical practice, with an objective response rate (ORR) reaching 60% (97, 98). Research has also shown that miRNAs participate in the modulation of paclitaxel resistance (**Table 2**). Addressing chemoresistance may further improve the ORR.

**Table 2:** miRNAs and paclitaxel resistance in NPC.

miRNA	Expression	Source	Targets or pathway
miR-BART7-3p	Up	Cell,Tissue	SMAD7
miR-130b-5p	Up	Cell	–
miR-422a	Down	Cell,Tissue	FOXQ1
miR-29c	Down	Cell	ITGB1
miR-29a	Down	Cell	STAT3

Wang et al. confirmed that when miR-130b-5p was upregulated, cell invasion or movement under the effect of EMT was enhanced, and the suppressive effect of NEF on metastasis and chemoresistance to Taxol was weakened (99). However, there is a close correlation between miR-422a overexpression and high survival in NPC patients. Furthermore, miR-422a suppressed its target gene, FOXQ1, and reduced the risk of EMT, metastasis and docetaxel tolerance to chemotherapy (100). According to a TdT-mediated dUTP-biotin nick end labeling (TUNEL) assay and the acquired ratio of BAX to BCL-2, excessive miR-29c expression and a decrease in ITGB1 enabled NPC cells to be more sensitive to paclitaxel and accelerated cell death (64). Transfection of the miR-29a mimic suppressed the expression of PCNA, STAT3 and p-STAT3; slowed cell proliferation; and accelerated cell death by suppressing Bcl-2 and STAT3. Moreover, there is a negative association between the expression level of miR-29a and drug resistance in NPC patients (101).

#### 3.3.3 5-Fluorouracil

5-Fluorouracil (5-Fu) is another traditional chemical applied for head and neck cancer treatment. Similarly, miRNAs influence 5-Fu resistance through various phenotypes (**Table 3**). It was reported that overexpressed miR-299 targeted VEGFA and inhibited NPC cell proliferation and invasion and promoted chemotherapeutic sensitivity to 5-Fu (102). Liu et al. reported that treating NPC cells with the ERRα inverse agonist XCT-790 or knocking down ERRα could reduce resistance to chemotherapy. Furthermore, when cells were transfected with the miR-137 mimic, ERRα mRNA became less stable, and NPC cell sensitivity to 5-Fu therapy improved. In the case of ERRα knockdown, the demand for glucose decreased in chemo resistant cells, and the generation rate of ATP and lactate decreased (103). Additionally, the tumor suppressor miRNA miR-133a-3p induces 5-Fu sensitivity and directly targets the AKT/PI3K/c-Myc/P53/EGF signaling pathway to reduce cell cycle arrest at the G1/S stage (104).

**Table 3:** miRNAs and 5-Fu resistance in NPC.

miRNA	Expression	Source	Targets or pathway
miR-BART7-3p	Up	Cell,Tissue	SMAD7
miR-515-5p	Down	Cerum	IL-25
miR-299	Down	Cell	VEGFA
miR-133a-3p	Down	Cell,Tissue	EGFR,PI3K/AKT
miR-137	Down	Cell	ERRα

#### 3.3.4 Other drugs

Xue et al. described the effect of miR-129 overexpression on promoting apoptosis in a subline of NPC cells resistant to an HDAC inhibitor (SAHA). Experiments on endogenous xenograft suggested that miR-129 targeting Bcl-2 can address the issue of NPC cell resistance to SAHA (105). Another study reported that when miR-BART19-3p and miR-BART17-5p mimics were combined to inhibit BRCA1 expression in NPC cells, the DNA repair ability was weakened, while the doxorubicin sensitivity of the cells was improved (106) (**Table 4**).

**Table 4:** miRNAs and other drugs resistance in NPC.

miRNA	Expression	Source	Targets or pathway
miR-BART17-5p,miR-BART19-3p	Down	Cell	BRCA1
miR-129	Down	Cell,Tissue	Bcl-2

## 4 Conclusions and perspectives

**Figure 3:**
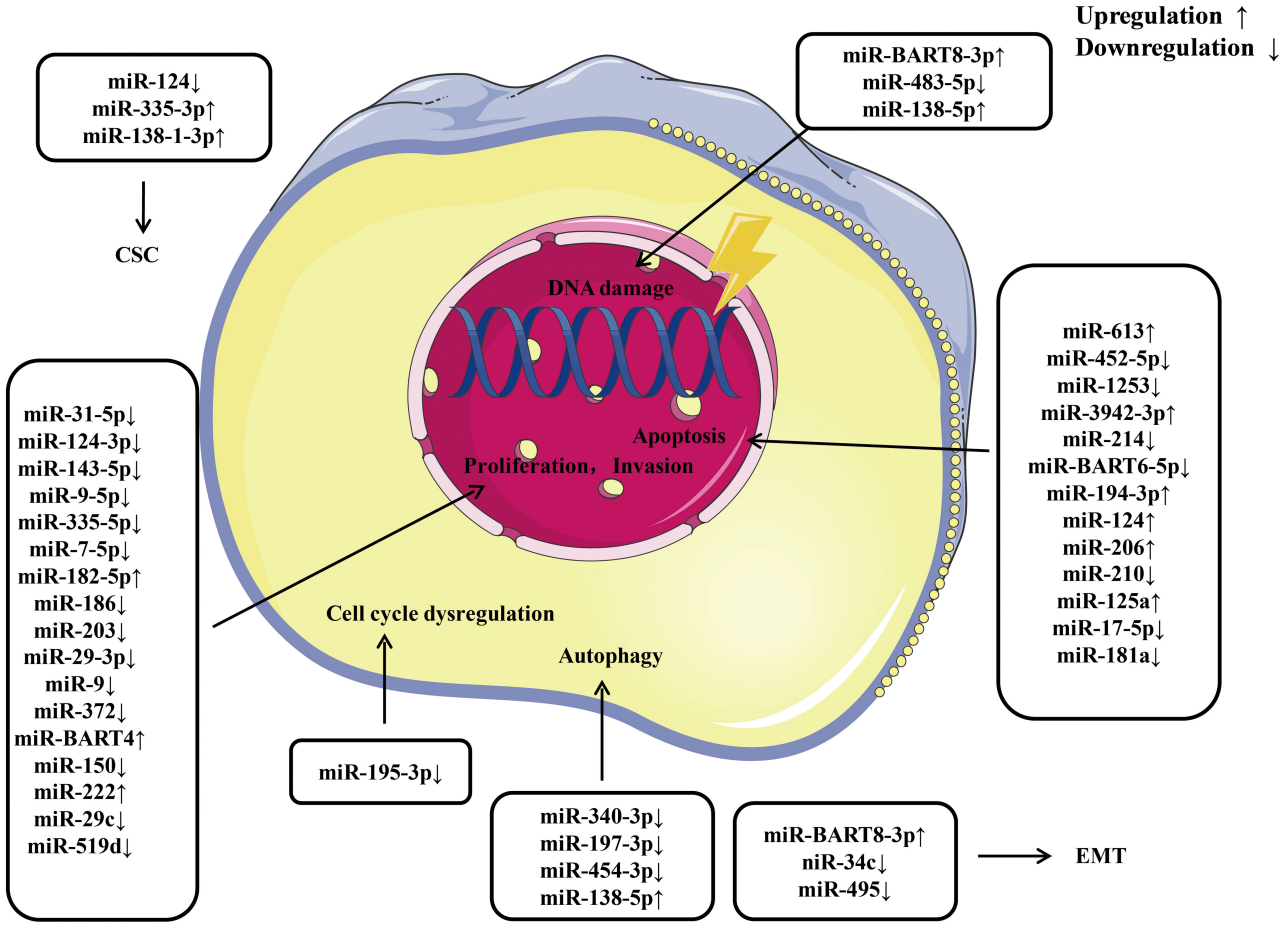
An overview of miRNAs implicated in the radio resistance of nasopharyngeal carcinoma. Various miRNAs involve in radio resistance of nasopharyngeal carcinoma by affecting cell proliferation, invasion, apoptosis, cell cycle, autophagy, epithelial-mesenchymal transition, and cancer stem cell via modulating the expression of downstream target gene. CSC, cancer stem cell; EMT, epithelial mesenchymal transition.

**Figure 4:**
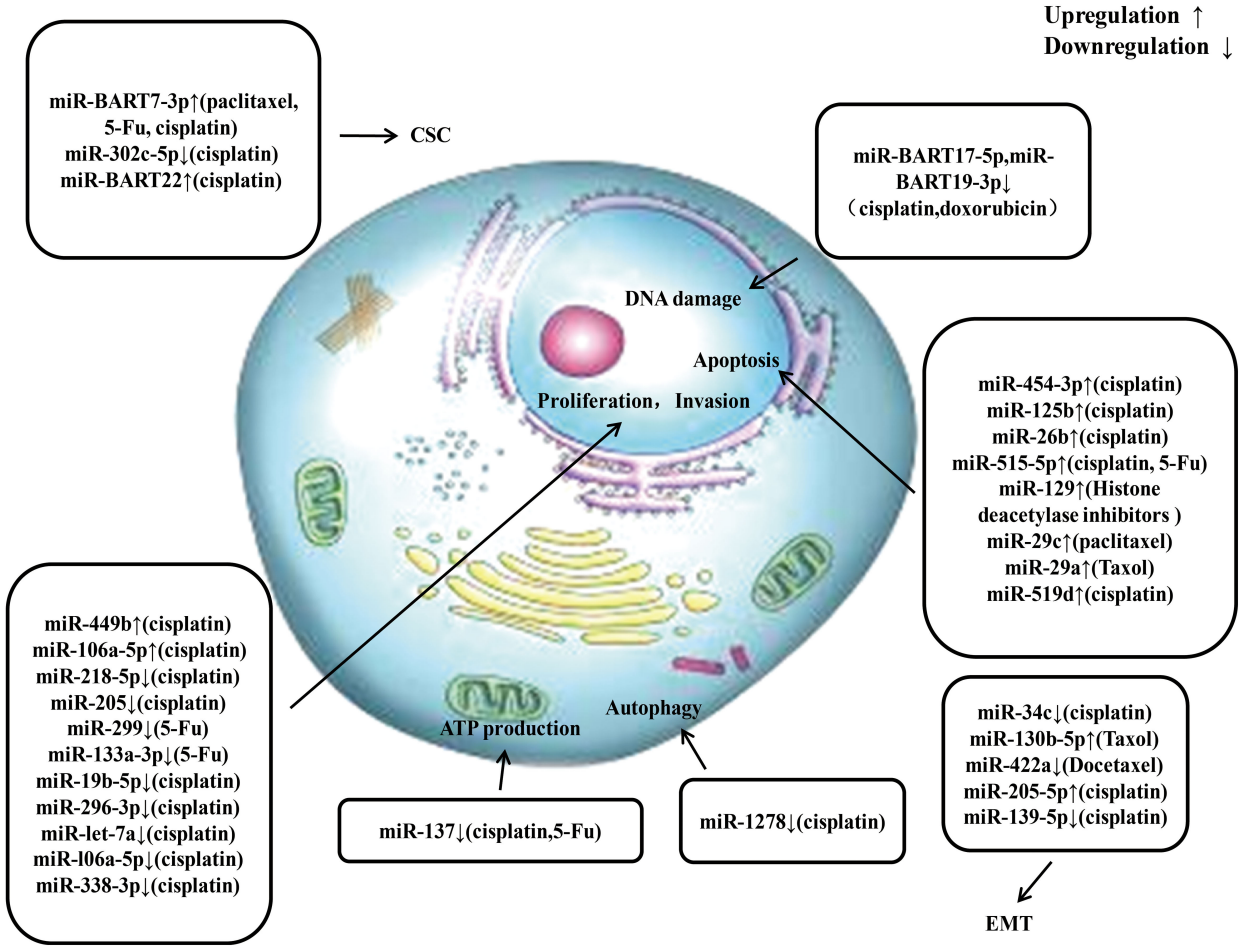
An overview of miRNAs involved in drug resistance of nasopharyngeal carcinoma. Multiple miRNAs participate in chemoresistance of nasopharyngeal carcinoma by affecting cell proliferation, invasion, apoptosis, ATP production, autophagy, epithelial-mesenchymal transition, and cancer stem cell via modulating the expression of downstream target gene. CSC, cancer stem cell; EMT, epithelial mesenchymal transition.

Currently, increasing amount of miRNAs have been found to significantly affect the radio resistance and chemoresistance of NPC. The potential detailed mechanisms by which miRNAs regulate radio resistance and chemoresistance in NPC are summarized in **Figures 3**, **4**, respectively. These endogenous dysregulated miRNAs can predict NPC progression and prognosis. Although targeting these dysregulated endogenous miRNAs has rarely been employed in NPC research, studies in other tumors have revealed the potential value of this strategy. For instance, natural drugs can regulate miRNA functions and nanoparticle carriers of synthetic oligonucleotides targeting cancerogenic miRNAs, or other valuable repressor miRNAs are now applied in liver cancer treatment. The specific mechanisms of certain miRNAs in regulating radio resistance or chemoresistance have not been determined. Therefore, screening key regulators from among the numerous candidate miRNAs is still challenging. An increasing number of clinical trials and translational studies are needed to identify the optimum NPC therapies based on miRNAs, which may ultimately lead to possible ways to overcome NPC chemoradiotherapy resistance. In addition to their canonical functions, the roles of unconventional miRNAs in chemoradiotherapy resistance are mainly unclear. Future advancements in this field of study may open up new avenues for treating chemoradiotherapy resistance.

## Author contributions

HX: Writing – original draft. WL: Supervision, Writing – review & editing. DW: Conceptualization, Funding acquisition, Resources, Writing – review & editing.
